# 1,3-Bisdiphenylethenyl-substituted Carbazolyl Derivatives as Charge Transporting Materials

**DOI:** 10.3390/molecules171214846

**Published:** 2012-12-13

**Authors:** Giedre Bubniene, Maryte Daskeviciene, Audrius Pukalskas, Vygintas Jankauskas, Vytautas Getautis

**Affiliations:** 1 Faculty of Chemical Technology, Kaunas University of Technology, Radvilenu pl. 19, Kaunas LT-50254, Lithuania; 2 Department of Solid State Electronics, Vilnius University, Sauletekio 9-III, Vilnius LT-10222, Lithuania

**Keywords:** carbazole, hydrazone, 1,3-indandione, ionization potential, charge carrier mobility

## Abstract

Synthesis of 1,3-diphenylethenylcarbazolyl-based charge transporting materials involving electron donating hydrazone moieties and an electron withdrawing 1,3-indandione moiety is reported. The obtained materials were examined by various techniques, including differential scanning calorimetry, UV-Vis spectroscopy, xerographic time of flight technique and the electron photoemission in air method. Photoemission spectra of the amorphous films of the investigated compounds showed ionization potentials of 5.54–5.90 eV. The hole drift mobility was measured by the xerographic time of flight technique. The highest hole drift mobility, exceeding 10^−5^ cm^2^/V·s at 6.4 × 10^5^ V/cm electric field, was observed for the 1,3-diphenylethenylcarbazolyl derivative molecularly doped with a *N,N*-diphenylhydrazone moiety in the polymeric host bisphenol-Z polycarbonate (PC-Z).

## 1. Introduction

Charge transporting materials (CTMs) are key components for the fabrication of high-performance electronic and optoelectronic devices using organic materials as active elements. Among CTMs carbazole-based compounds represent one of the most widely used and studied class of substances. Carbazole derivatives are well-known to exhibit good electro- and photo-active properties due to their high hole transporting mobility, strong absorption in the ultraviolet spectral region and blue-light emission. Since the discovery of photoconductivity in poly(9-vinylcarbazole) (PVK) [1] carbazole-containing derivatives became the subject of numerous investigations for applications in electrophotography [2]. The second wave of interest in carbazole-based CTMs is connected mostly with the discovery of organic light emitting diodes [3] and organic photorefractive materials [4]. Apart from electrophotographic photoreceptors [5], light-emitting diodes and photorefractive materials, carbazole-containing transporting materials are studied as the components of photovoltaic devices [6,7,8] and field-effect transistors [9].

Novel carbazole based derivatives possessing 1,3- or 3,6-diphenylethenyl fragments have been reported by us recently [10]. Commercial availability and relative cheapness of the starting materials, simple synthesis, number of sites available for easy functionalization, good charge drift mobility and solubility in common organic solvents makes these precursors attractive building blocks for the construction of more complex materials for optoelectronic applications. In the present work, synthesis of 1,3-diphenylethenylcarbazolyl-based CTMs containing electron donating hydrazone moieties and electron withdrawing 1,3-indandione moieties are reported. Optical, thermal, and photophysical properties of the synthesized carbazole derivatives are reported.

## 2. Results and Discussion

### 2.1. Synthesis

**Scheme 1:**
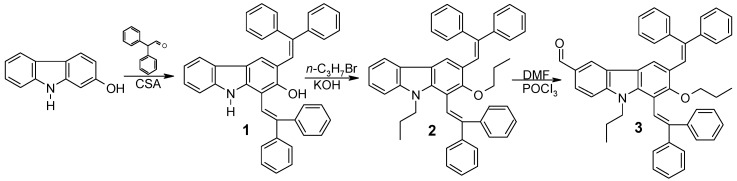
Synthesis route to the aldehyde **3**.

Recently we have showed that condensation of 9*H*-carbazol-2-ol with diphenylacetaldehyde in the presence of catalyst (±)-camphor-10-sulfonic acid (CSA, with the water generated during the course of the reaction being removed with 4 Å molecular sieves) results in formation of 1,3-bis(2,2-diphenylethenyl)-9*H*-carbazol-2-ol (**1**) [10]. In the present work, carbazol-2-ol derivative **1** (Scheme 1) was synthesized according to the modified procedure, using a mixture of toluene and dioxane (3/2, v/v) as solvent and water generated during the course of the reaction was removed using a Dean-Stark trap; such a procedure modification significantly reduces the duration of the reaction.

A simple alkylation with 1-bromopropane was performed to yield 1,3-bis(2,2-diphenylethenyl)-9-propyl-2-propoxycarbazole (**2**), which was used in Vilsmeier formylation to confirm that the 6-position of the carbazole remained active for electrophilic substitution reactions. The intermediate compound 1,3-bis(2,2-diphenylethenyl)-6-methanoyl-9-propyl-2-propoxycarbazole (**3**) was successfully isolated. The highest yield of aldehyde **3** was achieved when a large excess (6 equivalents) of the DMF/POCl_3_ complex was used.

**Scheme 2:**
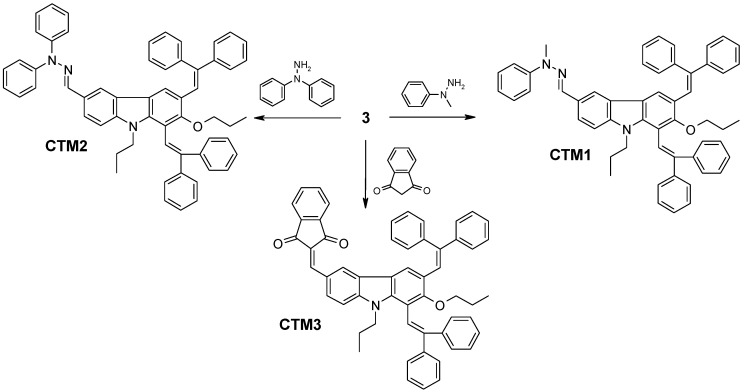
Synthesis routes to charge-transporting materials **CTM1**–**3**.

Finally, 1,3-bisdiphenylethenylcarbazolyl-based charge transporting materials **CTM1**, **CTM2** and **CTM3** were synthesized by the synthetic route shown in Scheme 2. Hole transporting materials containing electron donating hydrazone moieties **CTM1** and **CTM2** were prepared by interaction of aldehyde **3** with *N*-methyl-*N*-phenyl- or *N,N*-diphenylhydrazine, respectively. Meanwhile, in the reaction of aldehyde **3** with 1,3-indandione, a charge transporting material possessing both electron donating 1,3-bisdiphenylcarbazolyl moiety and an electron withdrawing 1,3-indandione moiety was synthesized. We expected that this potentially bipolar material could transport both holes and electrons as it comprises donor and acceptor segments, which demonstrate the good transporting properties of the appropriate charge carriers [11].

### 2.2. Thermal Properties

The obtained compounds are soluble in common organic solvents, such as THF, acetone, MEK, and toluene at room temperature. The thermal properties of the synthesized materials were determined by differential scanning calorimetry (DSC). The melting (*T_m_*) points and glass transition temperatures (*T_g_*) of the synthesized compounds are presented in Table 1. The data of the parent compound **2** was added for the comparison.

**Table 1:** Thermal parameters of **CTM1**–**3**.

Compound	2	CTM1	CTM2	CTM3
*T_m_* (°C)	144	239	220	239
*T_g_* (°C)	64	92	94	107

**Figure 1:**
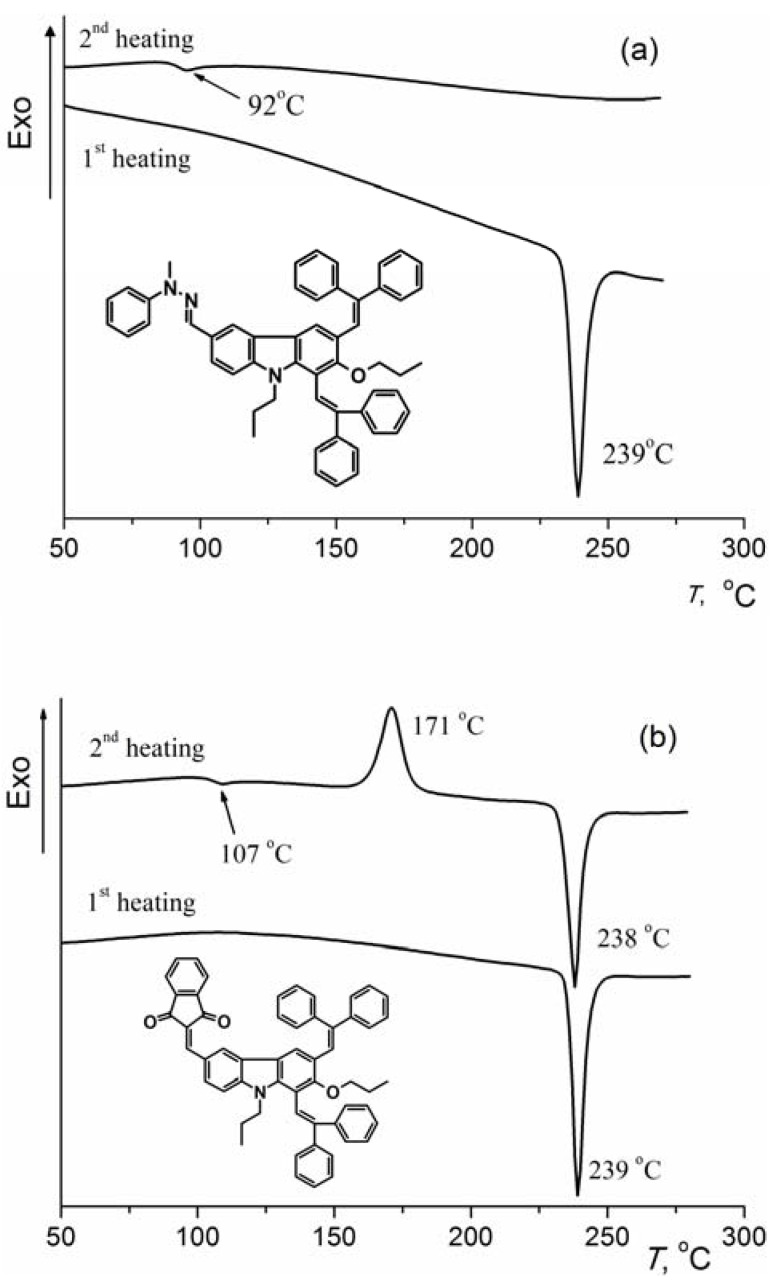
(**a**) DSC curves of **CTM1**; (**b**) DSC curves of **CTM3** (heating rate 10 K/min).

The DSC analysis of the **CTM1** reveals melting of the crystals at 239 °C during the first heating (Figure 1a). No recrystallization takes place during cooling or second heating, and only a glass transition at 92 °C is observed in the second heating, indicating that **CTM1** remains in glassy state after melting and subsequent cooling. This is common feature for other 1,3-bisdiphenylethenylcarbazolyl-based charge transporting material possessing hydrazone moieties investigated here. The glassy state of these materials is quite stable, and no signs of crystallization were detected during over year’s storage at ambient conditions. Meanwhile, indandione derivative **CTM3** demonstrates different behavior (Figure 1b). During the second heating a glass transition occurs at 107 °C, followed by crystallization at 171 °C and subsequent melting of the crystals with an endothermic peak at 238 °C. Such a DSC pattern indicates that the amorphous state of the **CTM3** isn’t stable and it recrystallizes easily.

*T*_g_ values of the obtained compounds demonstrate that the hydrazone and indandione fragments have a significant influence on the glass transition temperatures of the investigated derivatives. *T*_g_ was found to be higher for **CTM1**–**3** (92 °C for **CTM1**, 94 °C for **CTM2** and 107 °C for **CTM3**) than it was observed for the parent compound **2** (64 °C). Meanwhile, the *T*_g_ value for a hydrazone with the *N*,*N*-diphenyl moiety (**CTM2**) is very similar that of the hydrazone possessing an *N*-methyl-*N*-phenyl fragment (**CTM1**). The replacement of the hydrazone segment by the indandione moiety increases the glass transition temperature, but decreases the stability of amorphous state of the bipolar compound **CTM3**.

### 2.3. UV-Vis Spectra

**Figure 2:**
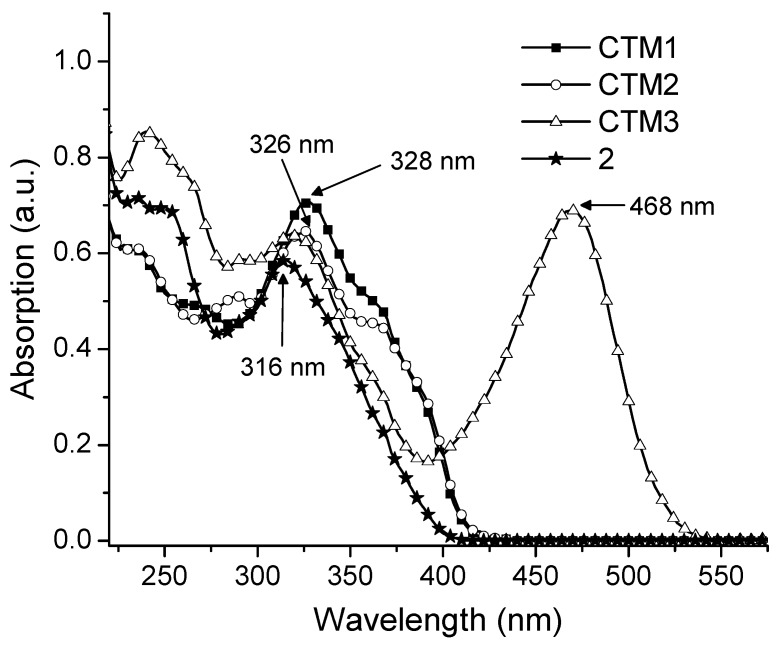
UV/Vis absorption spectra of 10^−4^ M THF solutions of **CTM1**–**3** and **2**.

Size of the conjugated *π*-electrons system is very important for the charge transporting process in the CTM structures, therefore, it was investigated using UV-Vis absorption spectroscopy. The absorption spectrum of the parent compound **2** was given for the comparison. Electron transitions to the higher energy states in the investigated derivatives give two main photon absorption maxima (Figure 2) at *ca.* 240 nm and 325 nm. The comparison of UV/Vis spectra of **CTM1** and **CTM2** with the parent compound **2** shows that the incorporation of hydrazone fragment into the structure of compound **2** doesn’t contribute too much to the existing π-conjugated system. The absorption spectra of both **CTM1** and **CTM2** are bathochromically shifted by just 8–10 nm. Also there isn’t much difference in the π→π * absorption band when comparing hydrazones **CTM1** and **CTM2** between each other. The replacement of the methyl group in **CTM1** by a phenyl group in **CTM2** leads to a slight additional bathochromic shift of 2 nm. In both cases this is most likely due to unfavorable arrangement of the fragments in the molecule.

Presence of the donor and acceptor moieties in the structure of **CTM3** results in the appearance of the strong absorption band around 468 nm due to the intramolecular charge transfer process (ICT). Due to this ICT compound **CTM3** has an intense orange color.

### 2.4. Photophysical Properties

**Figure 3:**
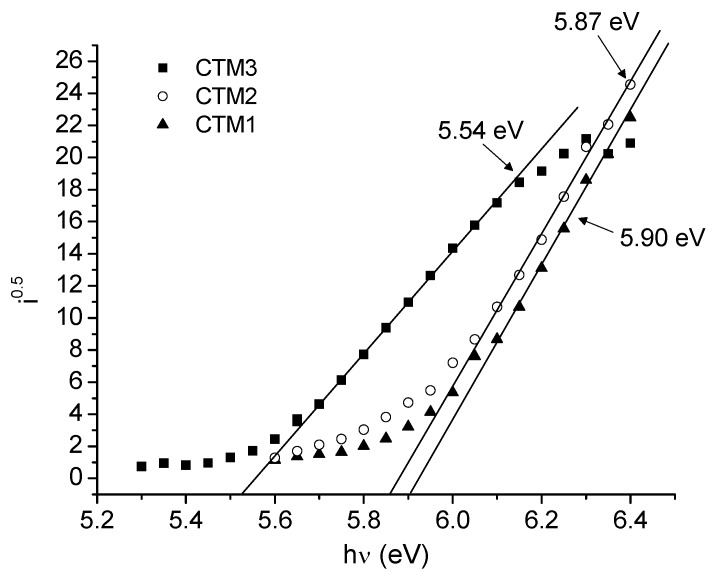
Photoemission spectra of the amorphous films of **CTM1**–**3** measured in air.

Photoemission spectra of the films of the investigated compounds **CTM1**–**3** are presented in Figure 3. The ionization potential values obtained by the electron photoemission in air method are presented in Table 2. Usually the photoemission experiments are carried out under vacuum and high vacuum is one of the main requirements for these measurements. If the vacuum is not high enough, the sample surface oxidation and gas adsorption influence the measurement results. In our case, however, the organic material investigated is stable enough to oxygen and the measurements may be carried out in air.

**Table 2:** *I_p_* and hole drift mobility data for mixtures of **2** and **CTM1**–**3** with PC-Z.

Compound	*I_p_* (eV)	Layer composition	Layer thickness (μm)	*μ*_o_ (cm^2^/V·s)	*μ* (cm^2^/V·s) ^1^
**CTM1**	5.90	Al+(**CTM1**+PC-Z, 1:1)	9	4·10^−7^	1.8·10^−5^
**CTM2**	5.87	Al+(**CTM2**+PC-Z, 1:1)	8	1.6·10^−7^	9·10^−6^
**CTM3**	5.54	Al+(**CTM3**+PC-Z, 1:1)	5.4	2·10^−8^	4·10^−6^

^1^ at 6.4 × 10^5^ V/cm electric field.

The measured ionization energies vary in the range from 5.54 eV to 5.90 eV. Introduction of hydrazone fragments significantly increases the ionization potential values by ~0.3 eV (compared to 5.6 eV for the parent compound **2**). This is somewhat unusual—to our knowledge this is the highest *I*_p_ values among hydrazone derivatives investigated as hole transporting materials [12]. The *I*_p_ for the bipolar compound **CTM3** possessing both electron donating 1,3-bisdiphenylcarbazolyl moiety and electron windrawing 1,3-indandione moieties is 5.54 eV.

**Figure 4:**
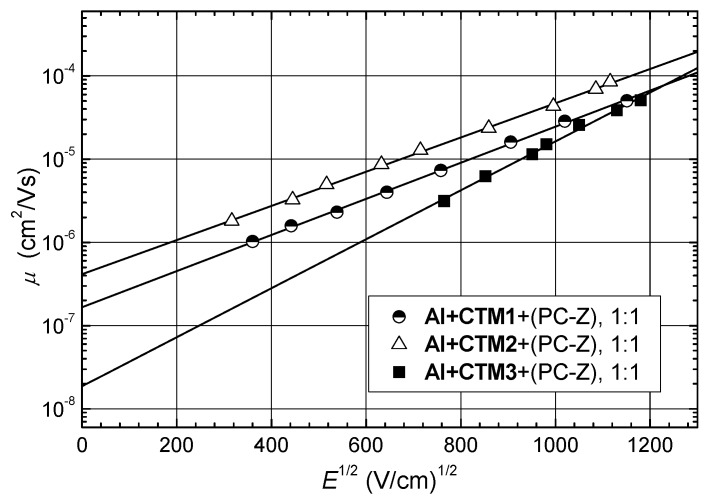
Electric field dependencies of the hole-drift mobilites (*μ*) in charge transport layers of compounds **CTM1**–**3** doped in PC-Z (mass proportion 1:1).

Crystalline CTMs usually are used for layer preparation in compositions based on a polymeric binder such as PC-Z. The charge transport properties of the synthesized compounds **CTM1**–**3** were studied by the xerographic time of flight technique. The zero field hole drift mobility parameter *μ*_o_ as well as the hole drift mobility *μ* value at the 6.4 × 10^5^ V/cm field strength are given in Table 2. Figure 4 shows the room temperature dependencies of the hole drift mobility on the electric field in the films of the investigated compounds molecularly doped in polymeric host PC-Z (50% solid solutions).

**Figure 5:**
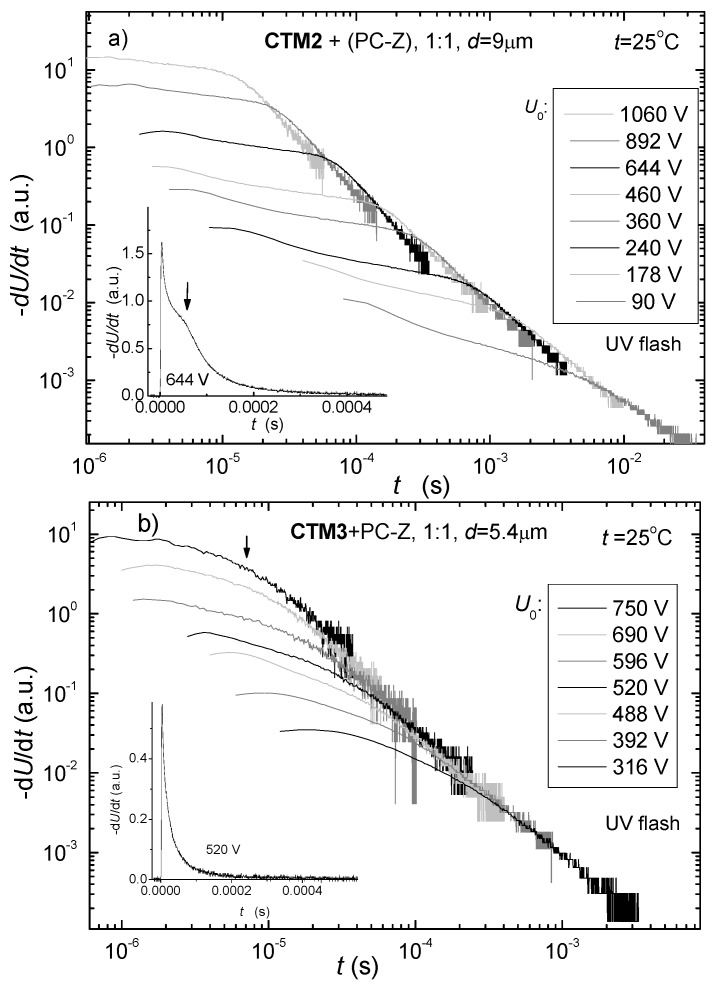
(**a**) XTOF transients for **CTM2** (1:1 composition with PC-Z); (**b**) XTOF transients for **CTM3** (1:1 composition with PC-Z).

Hole drift mobilities of the investigated 1,3-bisdiphenylethenyl-substituted carbazolyl derivatives, doped in PC-Z reach 10^−5^ cm^2^/Vs for strong electric fields. Comparing the charge carrier-transport properties of **CTM1** and **CTM2**, it is interesting to note that *N,N*-diphenylhydrazone derivative **CTM2** shows higher mobility than the *N*-methyl-*N*-phenyl analogue **CTM1**. The slightly extended conjugation network in compound **CTM2** most probably results in stronger electronic coupling between molecules, thereby facilitating hole hopping. The comparison of compound **CTM3** containing an indandione fragment, reveals that mobility values drop. On the other hand the hole mobility in **CTM3** is two orders of magnitude higher than in methylene-1,3-indandione derivatives reported in the literature [13]. The XTOF transients for the compound **CTM3**, were of disperse character, but the transit time was well seen on lg-lg plots (Figure 5a). Meanwhile, hole transport transients are with well-defined transit time on linear plots in case of **CTM1** and **CTM2** (Figure 5b).

Unfortunately, we were unable to measure the electron drift mobility of the bipolar compound **CTM3** because of the too dispersive charge carrier transport.

## 3. Experimental

### 3.1. General

9*H*-carbazol-2ol, diphenylacetaldehyde, (±)-camphor-10-sulfonic acid, indan-1,3-dione, *N*-methyl-*N*-phenylhydrazine and *N,N*-diphenylhydrazine hidrochloride were purchased from Aldrich Chemical Co. (Milwaukee, WI, USA) and were used without purification. 1,3-bis(2,2-Diphenylethenyl)-9-propyl-2-propoxycarbazole (**2**) and 1,3-bis(2,2-diphenyl-ethenyl)-6-methanoyl-9-propyl-2-propoxycarbazole (**3**) were obtained according to the methods described in [10]. The course of the reactions were monitored by TLC on ALUGRAM^®^ SIL/UV_254_ plates, using a mixture of acetone and *n*-hexane as eluent and developed with iodine or UV radiation. The ^1^H-NMR spectra were taken on a Varian Unity Inova (300 MHz) spectrometer in CDCl_3_. All the data are given as chemical shifts in *δ* (ppm), (CH_3_)_4_Si (TMS, 0 ppm) was used as an internal standard. The IR spectra of synthesized compounds were recorded in KBr pellets on a Perkin Elmer Spectrum BX II FT-IR System spectrometer. MS analysis was performed on a Maxis 4G Q-TOF instrument (Bruker Daltonik GmbH, Bremen, Germany). Compounds were ionized in ESI positive mode. Capillary voltage was set to 4,500 V, N_2_ gas was used as nebulizer at a pressure of 1.5 bar, and as drying gas at a flow rate of 8 L/min. Drying temperature was set at 200 °C. Sample was injected with a 6-port valve equipped with 20 µL sample loop. The eluent flow rate was 0.4 mL/min. No fragmentation was performed. Elemental analysis was performed with an Exeter Analytical CE-440 Elemental Analyzer. Melting points were determined using Electrothermal Mel-Temp melting point apparatus. The UV-vis spectra were recorded on a Perkin Elmer Lambda 35 spectrometer in THF (10^−4^ M) in a microcell with an internal width of 1 mm. The differential scanning calorimetry (DSC) measurements were carried out on a Mettler DSC 30 calorimeter at a scan rate of 10 K/min under nitrogen atmosphere. The glass transition temperatures (*T*_g_) were determined from the second heating.

### 3.2. Synthesis Procedures

*1,3-Bis(2,2-diphenylethenyl)-carbazol-2-ol* (**1**). 9*H*-Carbazol-2-ol (15.0 g, 0.082 mol) was dissolved in a mixture of toluene (75 mL) and 1,4-dioxane (50 mL), then (±)-camphor-10-sulfonic acid (19.04 g, 0.082 mol) was added and the mixture was heated at reflux for 20 min. Afterwards diphenylacetaldehyde (40.2 g, 0.205 mol) was added and reflux continued (2 h) using a Dean-Stark trap. After termination of the reaction (TLC control, acetone/*n*-hexane, 7:18), the mixture was treated with ethyl acetate and washed with distilled water. The organic layer was dried over anhydrous MgSO_4_, filtered and solvents were removed. The residue was dissolved in the mixture of toluene and 2-propanol (1/3, v/v). The crystals, formed upon standing in RT, were filtered off, washed with 2-propanol and dried. The yield of **1** was 25.0 g (57%) as a pale yellow powder, mp: 193–195 °C (toluene, 194–195 °C [10]). ^1^H-NMR δ 7.57–7.50 (m, 1H, NH); 7.45–6.96 (m, 27H, Ar); 5.31 (s, 1H, OH). ^13^C-NMR δ 150.5, 145.7, 143.5, 143.1, 142.9, 140.4, 139.68, 139.62, 137.4, 130.7, 129.8, 128.96, 128.74, 128.66, 128.49, 128.37, 128.09, 127.65, 126.18, 124.6, 123.8, 121.6, 120.5, 119.57, 119.36, 117.99, 116.74, 110.28, 107.41. IR ν (cm^−1^): 3537 (OH); 3440 (NH); 3077, 3056, 3028 (aromatic CH); 1625, 1611, 1594, 1490, 1444 (C=C, C–N); 1183 (C_Ht_–OH); 806, 779, 769, 760, 736, 701, 654 (CH=CH 1,2,3-trisubstituted carbazole and monosubstituted benzene). Anal. Calcd for C_40_H_29_NO: C 89.02; H 5.42; N 2.60. Found: C 88.90; H 5.53; N 2.45.

*1,3-Bis(2,2-diphenylethenyl)-6-(1-phenyl-1-methylhydrazon-2-yl)-9-propyl-2-propoxycarbazole* (**CTM1**). 1,3-bis(2,2-Diphenylethenyl)-6-methanoyl-9-propyl-2-propoxycarbazole (2, 1.0 g, 1.53 mmol) was dissolved in tetrahydrofuran (5 mL) and *N*-phenyl-*N*-methylhydrazine (0.19 g, 1.53 mmol) was added. The mixture was refluxed until all compound **2** has reacted. The crystals formed upon standing of the cooled reaction mixture, were filtered off, washed with 2-propanol and dried. The yield was 1.10 g (95%), mp 235–236 °C. ^1^H-NMR δ 7.76 (d, *J* = 1.3 Hz, 1H, 5-H carbazole), 7.69 (dd, *J*_1_ = 8.5 Hz, *J*_2_ = 1.5 Hz, 1H, 7-H carbazole), 7.60 (s, 1H, CH=N), 7.55–6.89 (m, 29H, Ar), 4.66–4.05 (m, 2H, NCH_2_), 4.01–3.66 (m, 2H, OCH_2_), 3.43 (s, 3H, NCH_3_), 1.86–1.63 (m, 4H, NCH_2_CH_2_, OCH_2_CH_2_), 0.92 (t, *J* = 7.4 Hz, 3H, N(CH_2_)_2_CH_3_), 0.79 (t, *J* = 7.4 Hz, 3H, O(CH_2_)_2_CH_3_). IR ν (cm^−1^): 3078, 3054, 3024 (aromatic CH), 2957, 2931, 2872 (aliphatic CH), 1591, 1568, 1501, 1486, 1467, 1443 (C=C, C–N), 1203 (C_Ht_–O), 1135, 1113, 1071 (C–O–C), 800, 772, 764, 747, 736, 699 (CH=CH of 1,2,3,6,9-pentasubstituted carbazole, monosubstituted benzene). MS (APCI^+^), *m/z*: 756 ([M+H]^+^). Anal. Calcd for C_54_H_49_N_3_O: C, 85.79; H, 6.53; N, 5.56. Found: C, 86.03; H, 6.74; N, 5.68.

*1,3-Bis(2,2-diphenylethenyl)-6-(1,1-diphenylhydrazon-2-yl)-9-propyl-2-propoxycarbazole* (**CTM2**). The title compound was prepared according to the same procedure as described for **CTM1**, except that *N,N*-diphenylhydrazine hydrochloride (0.73 g, 4 mmol) dissolved in methanol (10 mL) was added into the solution of compound **2** (2.0 g, 3.07 mmol) in THF (3 mL). After termination of the reaction (TLC control), the mixture was extracted with ethyl acetate and water. The organic layer was washed with distilled water until the wash water was neutral, dried over anhydrous Mg_2_SO_4_, ethyl acetate was removed and the residue was dissolved in acetone. The crystals formed upon standing were filtered off and washed with diethyl ether. The yield was 1.04 g (41%), mp 220–221 °C. ^1^H-NMR δ 7.75 (s, 1H, CH=N), 7.58–6.98 (m, 38H, Ar), 4.66–4.04 (m, 2H, NCH_2_), 4.00–3.69 (m, 2H, OCH_2_), 1.83–1.64 (m, 4H, NCH_2_CH_2_, OCH_2_CH_2_), 0.91 (t, *J* = 7.4 Hz, 3H, N(CH_2_)_2_CH_3_), 0.77 (t, *J* = 7.4 Hz, 3H, O(CH_2_)_2_CH_3_). IR ν (cm^−1^): 3077, 3055, 3021 (aromatic CH), 2964, 2932, 2875 (aliphatic CH), 1591, 1494, 1460, 1444 (C=C, C–N), 1204 (C_Ht_–O), 1093, 1069, 1056 (C–O–C), 812, 764, 698 (CH=CH of 1,2,3,6,9-pentasubstituted carbazole, monosubstituted benzene). MS (APCI^+^), *m/z*: 818 ([M+H]^+^). Anal. Calcd for C_59_H_51_N_3_O: C, 86.62; H, 6.28; N, 5.14. Found: C, 86.40; H, 6.15; N, 5.26.

*1,3-Bis(2,2-diphenylethenyl)-6-(indan-1,3-dione-2-methylen)-9-propyl-2-propoxycarbazole* (**CTM3**). A solution of aldehyde **2** (1.5 g, 2.3 mmol) and indan-1,3-dione (0.67 g, 4.6 mmol) in dioxane (8 mL) was heated at reflux for 48 h. After termination of the reaction (TLC control), the product was isolated similarly as compound **3**. The yield was 1.3 g (72%), mp 238–239 °C. ^1^H-NMR δ 8.86 (dd, *J*_1_ = 8.8 Hz, *J*_2_ = 1.7 Hz, 1H, 7-H carbazole), 8.47 (d, 1H, *J* = 1.6 Hz, 5-H carbazole), 8.06–7.95(m, 3H, Ar), 7.84–7.74 (m, 2H, indan-1,3-dione), 7.53–6.98 (m, 24H, Ar), 4.74–4.13 (m, 2H, NCH_2_), 4.04–3.69 (m, 2H, OCH_2_), 1.89–1.67 (m, 4H, NCH_2_CH_2_, OCH_2_CH_2_), 0.93 (t, *J* = 7.4 Hz, 3H, N(CH_2_)_2_CH_3_), 0.83 (t, *J* = 7.4 Hz, 3H, O(CH_2_)_2_CH_3_). IR ν (cm^−1^): 3078, 3054, 3022 (aromatic CH), 2957, 2929, 2874 (aliphatic CH), 1718, 1675 (C=O), 1629, 1596, 1575, 1558, 1488, 1464, 1443 (C=C, C–N), 1336 (C=O), 1229, 1092, 1068 (C–O–C), 1200 (C_Ht_–O); 816, 769, 737, 698 (CH=CH of 1,2,3,6,9-pentasubstituted carbazole, monosubstituted benzene). MS (ESI^+^), *m/z*: 780 ([M+H]^+^). Anal. Calcd for C_56_H_45_NO_3_: C, 86.24, H, 5.82, N, 1.80. Found: C, 86.02; H, 5.96; N, 1.69.

### 3.3. Measurements

#### 3.3.1. Ionization Potential Measurement

Ionization potential (*I*_p_) was measured by the electron photoemission in air method described in [14,15]. The samples for the ionization energy measurement were prepared by dissolving materials in THF and then coating them on Al plates precoated with ~0.5 μm thick methylmethacrylate and methacrylic acid copolymer adhesive layer. The thickness of the transporting material layer was 0.5–1 μm. The samples were illuminated with monochromatic light from the quartz monochromator with a deuterium lamp. The power of the incident light beam was (2–5) × 10^−8^ W. The negative voltage of *I*^0.5^ and *hν* near the threshold. The linear part of this dependence was extrapolated to the *hν* axis and the *I*_p_ value was determined as the photon energy at the interception point. The samples for hole drift mobility measurements were prepared from 1:1 mass proportion compositions of compounds with PC-Z (Iupilon Z-200 from Mitsubishi Gas Chemical Co. Tokyo, Japan). The sample substrate was polyester film with conductive Al layer. The layer thickness was in the range of 5–12 μm.

#### 3.3.2. Charge Transport Measurement

The sample for the measurement was prepared by drop casting of the solution of CTM in THF (100 mg in 1 mL) onto a polyester film with a conductive Al layer. After coating the sample was heated at 70 °C for 1 h. Thus the transporting layer of the sample was prepared. The thickness of the transporting layer was ~5–9 μm. The hole drift mobility was measured by xerographic time of flight technique (XTOF) [16]. Positive corona charging created electric field inside the transporting material (TM) layer. The charge carriers were generated at the layer surface by illumination with pulses of nitrogen laser (pulse duration was 2 ns, wavelength 337 nm). The layer surface potential decrease as a result of pulse illumination was up to 1–5% of the initial potential before illumination. The capacitance probe that was connected to the wide frequency band electrometer measured the rate of the surface potential decrease, *dU/dt*. The transit time *t*_t_ was determined by the kink on the curve of the *dU/dt* transient in linear scale. The drift mobility was calculated by the formula *μ* = *d*^2^/*U*_0_*t*_t_, where *d* is the layer thickness and *U*_0_ is the surface potential at the moment of illumination.

## 4. Conclusions

1,3-Bisdiphenylethenyl-substituted carbazolyl derivatives containing electron donating hydrazone moieties and an electron withdrawing 1,3-indandione moiety were synthesized and investigated as potential charge transporting materials for optoelectronics. It was determined that compounds with hydrazone moieties can function as hole transporting materials. The hole mobilities in the amorphous films of the investigated compounds **CTM1** and **CTM2** molecularly doped in the polymeric host PC-Z (50% solid solutions) reach 10^−5^ cm^2^/V·s in strong electric fields. It is intriguing that these hole TMs show very high *I*_p_ values (*ca*. 5.9 eV). The investigated indandione derivative **CTM3** exhibits dispersive charge carrier transport. The molecular structures of the synthesized 1,3-bisdiphenylethenyl substituted carbazolyl derivatives **CTM1** and **CTM2** allow them to exist in a stable amourphous state with glass transition temperatures of 92 °C and 94 °C, respectively.
